# Yes-associated protein (YAP) is a negative regulator of chondrogenesis in mesenchymal stem cells

**DOI:** 10.1186/s13075-015-0639-9

**Published:** 2015-05-30

**Authors:** Alexandra Karystinou, Anke J Roelofs, Anna Neve, Francesco P Cantatore, Henning Wackerhage, Cosimo De Bari

**Affiliations:** Musculoskeletal Research Programme, Institute of Medical Sciences, University of Aberdeen, Foresterhill, Aberdeen, AB25 2ZD UK; Rheumatology Clinic, Department of Medical and Surgical Sciences, University of Foggia, Via Napoli 25, 71122 Foggia, Italy

## Abstract

**Introduction:**

The control of differentiation of mesenchymal stromal/stem cells (MSCs) is crucial for tissue engineering strategies employing MSCs. The purpose of this study was to investigate whether the transcriptional co-factor Yes-associated protein (YAP) regulates chondrogenic differentiation of MSCs.

**Methods:**

Expression of total YAP, its paralogue transcriptional co-activator with PDZ-binding motif (TAZ), and individual YAP transcript variants during *in vitro* chondrogenesis of human MSCs was determined by quantitative reverse transcription polymerase chain reaction (RT-PCR). YAP expression was confirmed by western blotting. To determine the effect of high YAP activity on chondrogenesis, C3H10T1/2 MSC-like cells were transduced with human (h)YAP and treated in micromass with bone morphogenetic protein-2 (BMP-2). Chondrogenic differentiation was assessed by alcian blue staining and expression of chondrocyte-lineage genes. BMP signalling was determined by detection of pSmad1,5,8 by western blotting and expression of BMP target genes by quantitative RT-PCR. Finally, YAP and pYAP were detected in mouse embryo hindlimbs by immunohistochemistry.

**Results:**

YAP, but not TAZ, was downregulated during *in vitro* chondrogenesis of human MSCs. One of the YAP transcript variants, however, was upregulated in high-density micromass culture. Overexpression of hYAP in murine C3H10T1/2 MSCs inhibited chondrogenic differentiation. High YAP activity in these cells decreased Smad1,5,8 phosphorylation and expression of the BMP target genes Inhibitor of DNA binding/differentiation (Id)1, Id2 and Id3 in response to BMP-2. In developing mouse limbs, Yap was nuclear in the perichondrium while mostly phosphorylated and cytosolic in cells of the cartilage anlage, suggesting downregulation of Yap co-transcriptional activity during physiological chondrogenesis *in vivo*.

**Conclusions:**

Our findings indicate that YAP is a negative regulator of chondrogenic differentiation of MSCs. Downregulation of YAP is required for chondrogenesis through derepression of chondrogenic signalling. Therapeutic targeting of YAP to promote cartilage repair and prevent secondary osteoarthritis is an exciting prospect in rheumatology.

**Electronic supplementary material:**

The online version of this article (doi:10.1186/s13075-015-0639-9) contains supplementary material, which is available to authorized users.

## Introduction

Symptomatic joint surface defects require treatment to achieve repair and attempt prevention of secondary osteoarthritis (OA). Biological repair of the joint surface is becoming a clinical reality. Autologous chondrocyte implantation remains the gold standard of cell therapy for cartilage repair; however, chondrocyte preparations are known to be difficult to manufacture robustly because chondrocytes in culture have a limited lifespan and undergo de-differentiation, thereby losing their ability to form cartilage [1,2].

Mesenchymal stromal/stem cells (MSCs), present in bone marrow [3] and connective tissues such as periosteum [4,5] and synovium [6,7], are attractive alternative cells for the repair of articular cartilage due to their easy access and culture expansion and their capacity to form cartilage [8]. To fully harness the therapeutic value of these cells for cartilage repair, an in-depth understanding of the molecular regulation of chondrogenesis is essential.

Yes-associated protein (YAP; gene symbol *YAP1*) is a key transcriptional co-factor that has been implicated in recent years in the regulation of stem cell fate [9]. YAP and its paralogue transcriptional co-activator with PDZ-binding motif (TAZ) shuttle between the cytoplasm and the nucleus and interact with transcription factors to regulate their activity. Uncontrolled activity of YAP causes tissue overgrowth due to modulation of stem cell proliferation in multiple tissues and organs, including liver [10,11], intestine [11], brain [12], and epidermis [13], and we have shown that YAP increases proliferation in muscle satellite cells [14].

YAP is regulated by the Hippo pathway, comprising the kinases Mst1/2 (mammalian Ste20-like; a class II GC kinase) and Lats1/2 (large tumour suppressor; an Ndr kinase). Activation of the Hippo pathway, for example through cell-cell contact [15] or GPCR signalling [16], leads to phosphorylation of YAP on specific serine residues, most notably Ser127. Phosphorylation at Ser127 promotes its cytosolic retention and proteasomal degradation [10,17]. In addition, YAP is regulated by actomyosin cytoskeletal tension, thereby acting as transducer of mechanical cues exerted by extracellular matrix (ECM) stiffness and cell shape, with a stiff ECM and cell spreading increasing YAP activity [18].

YAP and its paralogue TAZ have been shown to be key factors in the regulation of MSC lineage commitment, with low YAP/TAZ activity promoting adipogenesis, while high YAP/TAZ activity drives MSCs towards osteogenesis [19-21]. The role of Yap in chondrogenesis is less clear and the mechanism of how YAP modulates chondrogenesis is not known.

Chondrogenic differentiation of MSCs is regulated by numerous hormones and growth factors [22]. The most prominent growth factors controlling chondrogenesis include the bone morphogenetic proteins (BMPs) and transforming growth factor (TGF) β, which signal via heteromeric receptors to induce phosphorylation of receptor-activated (R)-Smad 1,5, and 8, and R-Smad 2 and 3, respectively. Phosphorylated R-Smads form complexes with Smad4 and translocate to the nucleus, where they regulate transcription of a range of target genes such as the Inhibitor of DNA binding/differentiation (Id) family of proteins. BMP/TGF-β signalling is dampened by inhibitory Smad 6 and 7. YAP can modulate BMP/TGF-β signalling through direct interactions with both R-Smads and inhibitory Smads [23,24].

In the present study, we sought to clarify the role of YAP in the regulation of chondrogenesis of MSCs, hypothesising that YAP mediates its effects on MSC chondrogenesis through modulation of BMP/TGF-β signalling.

## Methods

### Cell isolation and culture

Human MSCs were obtained according to the declaration of Helsinki with ethical approval from the North of Scotland Research Ethics Committee. MSCs from human synovial membrane (hSM) or periosteum were isolated from specimens obtained post-mortem in the Laboratory for Skeletal Development and Joint Disorders, University of Leuven, Belgium, or following joint arthroplasty for OA after informed consent from patients, and expanded up to passage (p)3 as previously described [6,25], then routinely seeded at 2,000 cells/cm^2^. In addition, MSCs were isolated from human bone marrow, after consent from donors, in the Laboratory for Skeletal Development and Joint Disorders, University of Leuven, Belgium, or from iliac crest-derived bone marrow mononuclear cells purchased from Stemcell Technologies (Vancouver, BC, Canada), and routinely seeded at 3,000 cells/cm^2^. Human MSCs were used for experiments up to p8. The murine MSC-like cells (embryonic fibroblast line, C3H10T1/2 clone 8) were purchased from the American Type Culture Collection (Manassas, VA, USA), and maintained in alpha minimum essential medium (αMEM) supplemented with 4 mM L-glutamine and 10% foetal bovine serum (FBS).

### Chondrogenesis assay

*In vitro* chondrogenesis was performed using micromass culture by seeding 4 × 10^5^ cells in 20 μl droplets, as previously described [25]. Chondrogenic differentiation of human MSCs was induced by treatment with 10 ng/ml of transforming growth factor β1 (TGF-β1; Gibco, Life Technologies, Paisley, UK) in a chemically defined serum-free medium starting 24 h after seeding [25]. In mouse C3H10T1/2 cells, chondrogenesis was induced by treatment with recombinant human BMP-2 (Source Bioscience, Nottingham, UK) at 300 ng/ml in Dulbecco’s modified Eagles’s medium (DMEM) (4.5 g/l glucose) in the presence of 10% FBS and 50 μg/ml ascorbic acid [26], or 10 ng/ml of TGF-β1 in DMEM (4.5 g/l glucose) in the presence of 10% FBS, starting 3 h after cell seeding. Micromass cultures were analysed 7 days after seeding, unless otherwise indicated.

### Plasmids and retroviral transduction

hYAP1, hYAP1(S127A), hYAP2, and hYAP2(S127A) cDNAs were sub-cloned from bacterial expression vectors (Addgene (Cambridge, MA, USA) plasmids 17791, 17790, 17793 and 17794, respectively; [27]) into pMSCV-IRES-eGFP plasmids [28], as previously described [14]. Retroviruses were packaged in HEK293T cells using standard methods, and C3H10T1/2 cells (seeded the previous day at 15,000 cells/cm^2^) were incubated with viral supernatant in the presence of 4 μg/ml polybrene for 4 h. Transduction efficiency was monitored by eGFP fluorescence, and was typically >90% as determined by flow cytometry.

### BMP-2 treatment

To determine the effects of YAP on BMP signalling, C3H10T1/2 cells were seeded in micromass (4 × 10^5^ cells in 20 μl) and cultured overnight in DMEM (4.5 g/l glucose) supplemented with 1 mg/ml recombinant human insulin, 0.55 mg/ml transferrin, 0.5 ug/ml sodium selenite, 50 mg/ml bovine serum albumin (BSA), and 470 ug/ml linoleic acid (ITS+). The next day, medium was replaced with medium containing 300 ng/ml BMP-2 (dissolved in 20 mM acetic acid, pH 3.2) or vehicle, and protein or RNA was extracted 0.5 to 8 h later.

### RNA extraction, cDNA synthesis, and quantitative polymerase chain reaction (PCR)

Total RNA was extracted using TRIzol reagent (Invitrogen, Paisley, UK) according to standard protocols, and RNA was quantified using a NanoDrop ND-1000 spectrophotometer (Labtech, Uckfield, UK). cDNA was synthesised from up to 2 μg total RNA using random hexamer primers and SuperScript II Reverse Transcriptase (Invitrogen), according to the manufacturer’s instructions. Quantitative PCR (qPCR) was performed with a Roche LightCycler 480 using Taqman Probes Master (Roche, Basel, Switzerland) for Sox9, Col2a1 and Col10a1, or SYBR Green Master (Roche) for all other assays, according to the manufacturer’s instructions. Amplification of a single product of correct size was confirmed by agarose gel electrophoresis and/or melting curve analysis. Relative concentrations were quantified using a serially diluted standard curve of unknown target concentration, or calculated using the Pfaffl method [29], and normalised to expression of GAPDH or ACTB. Results were expressed as relative change from appropriate control or baseline. Primers were designed using Primer-BLAST (National Center for Biotechnology Information) or Universal ProbeLibrary software (Roche). Primer sequences (5′ to 3′) that were used are: hYAP-Fw1: CCTCTTCCTGATGGATGGGAAC; hYAP-Fw2: ACTCGGCTTCAGCCATGAAC; hYAP-Fw3: AGCCCACTCGGGATGTAACTTGA; hYAP-Fw4: ACCTGATGATGTACCTCTGCC; hYAP-Rev1: TATTCCGCATTGCCTGCCG; hYAP-Rev2: AGGGCTAACTCCTGCCGAA; hYAP-Rev3: CTGGTGGGGGCTGTGACGTT; hYAP-Rev4: CTAACTCCTGTGGCCTCACCT; hYAP-Rev5: ATTGCCTGTGGCCTCACCT; hYAP-Rev6: CCACTGTTAAGGAAAGGATCTG. hTAZ-Fw: ATCCCAGCCAAATCTCGTG; hTAZ-Rev: TTCTGCTGGCTCAGGGTACT; hCTGF-Fw: CCTGCAGGCTAGAGAAGCA; hCTGF-Rev: GATGCACTTTTTGCCCTTCT; hCYR61-Fw: AAGAAACCCGGATTTGTGAG; hCYR61-Rev: GCTGCATTTCTTGCCCTTT; hGAPDH-Fw: AACAGCGACACCCACTCCTC; hGAPDH-Rev: CATACCAGGAAATGAGCTTGACAA; mSox9-Fw: CAGCAAGACTCTGGGCAAG; mSox9-Rev: TCCACGAAGGGTCTCTTCTC; mCol2a1-Fw: ACCCCCAGGTGCTAATGG; mCol2a1-Rev: AACACCTTTGGGACCATCTTT; mCol10a1-Fw: GCATCTCCCAGCACCAGA; mCol10a1-Rev: CCATGAACCAGGGTCAAGAA; mId1-Fw: GAGTCTGAAGTCGGGACCAC; mId1-Rev: GATCGTCGGCTGGAACAC; mId2-Fw: ACAGAACCAGGCGTCCAG; mId2-Rev: AGCTCAGAAGGGAATTCAGATG; mId3-Fw: CATAGACTACATCCTCGACCTTCA; mId3-Rev: CACAAGTTCCGGAGTGAGC; mActb-Fw: CTAAGGCCAACCGTGAAAAG; mActb-Rev: ACCAGAGGCATACAGGGACA. Total YAP was detected using primers hYAP-Fw4 and hYAP-Rev6. Individual YAP transcript variants were detected using primers indicated in Table 1.

**Table 1:** **List of human YAP transcript variants with alternate names, primers used for detection by quantitative RT-PCR, and expected amplicon size(s) of PCR products**

**Transcript**		**Alternate names**			**Primers** ^**5**^		**Amplicon**
**No.**	**Accession** ^**1**^	**Komuro** ^**2**^	**Gaffney** ^**3**^	**Ensembl** ^**4**^	**Fw**	**Rev**	**(bases)**
1	NM_001130145.2	n/a	YAP2γ	YAP-002	1	1	307
2	NM_006106.4	n/a	YAP1α	YAP-001	2	2	205
3	NM_001195044.1	YAP2	YAP2α	YAP-005	1	2	305
4	NM_001195045.1	n/a	n/a	YAP-004	3	3	548
5	NM_001282098.1	YAP1	YAP1ß	YAP-201	2	4	213
6	NM_001282097.1	n/a	YAP1γ	n/a	2	1	207
7	NM_001282099.1	n/a	YAP1δ	n/a	2	5	211
8	NM_001282100.1	n/a	YAP2ß	YAP-202	1	4	313
9	NM_001282101.1	n/a	YAP2δ	YAP-203	1	5	311

^1^NCBI accession number; [45]; accessed on 6 Aug 2014; ^2^Komuro *et al*. [27]; ^3^Gaffney *et al*. [30]; ^4^[46]; ENSG00000137693; accessed on 6 Aug 2014; ^5^see Methods for details. RT-PCR, reverse transcription PCR; YAP, Yes-associated protein. Fw, forward; Rev, reverse.

### Protein extraction and western blotting

For detection of YAP, pYAP and green fluorescent protein (GFP), cells were lysed in 50 mM Tris–HCl containing 1 mM ethylenediaminetetraacetic acid (EDTA), 1 mM ethylene glycol tetraacetic acid (EGTA), 1% (v/v) Triton X-100, 2% (v/v) protease inhibitor cocktail (Sigma-Aldrich, St Louis, MO, USA), 10 mM β-glycerophosphate, 50 mM sodium fluoride (NaF), and 0.5 mM sodium orthovanadate (Na_3_Vo). For detection of phosphorylated Smad (pSmad)1,5,8 and total Smad1, cells were lysed in phosphate-buffered saline (PBS) containing 1 mM EDTA, 1% (v/v) Triton-X-100, 1% (v/v) nonidet P-40, 0.1% (w/v) sodium dodecyl sulphate (SDS), 0.5% (w/v) sodium deoxycholate, 20 mM NaF, 5 mM Na_3_Vo, 1% (v/v) protease inhibitor cocktail (Sigma-Aldrich), and 0.4% (v/v) phosphatase inhibitor cocktail 2 (Sigma-Aldrich). Equal protein amounts, as determined by bicinchoninic acid (BCA) protein assay (Sigma-Aldrich), were electrophoresed under reducing conditions on 12% polyacrylamide-SDS gels (Criterion™ XT precast gels; Bio-Rad, Hercules, CA, USA) and transferred onto polyvinyl difluoride membranes by semi-dry transfer. Membranes were incubated with the following primary antibodies overnight at 4°C: anti-YAP (Cell Signaling Technology (Danvers, MA, USA) #4912), anti-phosphorylated YAP (pYAP) (Ser127) (Cell Signaling Technology #4911), anti-GFP (Abcam (Cambridge, UK) #13970), anti-β-actin (Cell Signaling Technology #4967), anti-pSmad1,5,8 (Cell Signaling Technology #9511), or anti-Smad1 (R&D Systems (Minneapolis, MN, USA) #AF2039). YAP, pYAP and GFP were detected with horseradish peroxidase (HRP)-conjugated secondary antibodies followed by incubation with SuperSignal West Dura chemiluminescence substrate (Thermo Fisher Scientific, Waltham, MA, USA) on a Fluor-S MultiImager (Bio-Rad). pSmad1,5,8 and total Smad1 were detected by incubation with IRDye800 and Alexa Fluor 680-conjugated secondary antibodies, respectively, and analysis on a LI-COR Odyssey Infrared Imager (LI-COR Biosciences, Lincoln, NE, USA). Quantification was performed using LI-COR Odyssey Software v2.1.

### Alcian blue staining

Deposition of highly sulphated glycosaminoglycans was detected by whole-mount staining of micromasses with 0.5% of alcian blue 8 GS dye (Carl Roth, Karlsruhe, Germany) at pH 0.2, as described previously [25]. For relative quantitation, glycosaminoglycans were extracted from stained micromasses with 6 M guanidine HCl for 6 hours at room temperature, and absorbance was measured at 630 nm on a BioTEK™ plate-reader with Gen5 v1.05.11 software (BioTEK, Winooski, VT, USA). To normalise for DNA content, DNA was extracted from parallel micromasses by overnight incubation at 55°C in 50 mM Tris (pH 8.0) containing 100 mM EDTA, 100 mM NaCl, and 1% SDS, followed by precipitation using isopropanol, resuspension in 10 mM Tris (pH 8.0) containing 0.1 mM EDTA, and quantification using a NanoDrop ND-1000 spectrophotometer (Labtech).

### Cell proliferation

C3H10T1/2 cells, non-transduced or transduced with hYAP or empty vector retrovirus, were plated at 5,000 cells/cm^2^ (sub-confluent) or 25,000 cells/cm^2^ (confluent), and left to adhere overnight. The next day, medium was removed, cells were washed with PBS, and medium with or without 10% FBS was added to the cells. To measure cell proliferation after 24 h, cells were incubated with 10 μM EdU for 3.5 hrs, and EdU incorporation into replicating DNA was detected using a Click-iT EdU detection kit (Invitrogen) and analysis on a BD LSRII flow cytometer (BD Biosciences, Franklin Lakes, NJ, USA). The percentage of EdU-positive cells was quantified using FlowJo v7.6.1 software (Tree Star, Ashland, OR, USA).

### Immunohistochemistry

Animal experiments were approved by the UK Home Office and carried out in accordance with UK Home Office guidelines. C57Bl/6 mouse embryos at embryonic day 13.5 (E13.5), E14.5 and E16.0 were used for experiments. Embryos were fixed in 4% paraformaldehyde, and hindlimbs were dissected, dehydrated, embedded in paraffin, and cut into 5-μm thin sections using a microtome. Immunohistochemistry (IHC) using antibodies against YAP (Novus Biologicals, Littleton, CO, USA #NB110-58358), pYAP (Cell Signaling Technology #4911), or isotype control antibody, was performed as previously described [7]. Antigen retrieval was performed by boiling in a citrate-buffered antigen unmasking solution at pH6 (Vector Laboratories, Burlingame, CA, USA) in a waterbath for 20 min (YAP staining) or by incubation with 0.5 mg/ml porcine pepsin in 0.2 N HCl for 15 min at 37°C (pYAP staining). Sections were counterstained with haematoxylin. Tile scans were obtained using a Zeiss Axioscan Z1 slide scanner (Carl Zeiss, Oberkochen, Germany). Higher magnification images were obtained using an Axioskop 40 (Carl Zeiss) microscope with ProgRes C14 camera.

### Statistical analysis

Data was analysed using GraphPad Prism (GraphPad Software, San Diego, CA, USA). Gene expression in human MSCs was analysed by unpaired *t* test. All other data was analysed by two-way analysis of variance (ANOVA) with Bonferroni *post hoc* test.

## Results

### YAP is downregulated during chondrogenic differentiation of MSCs

**Figure 1:**
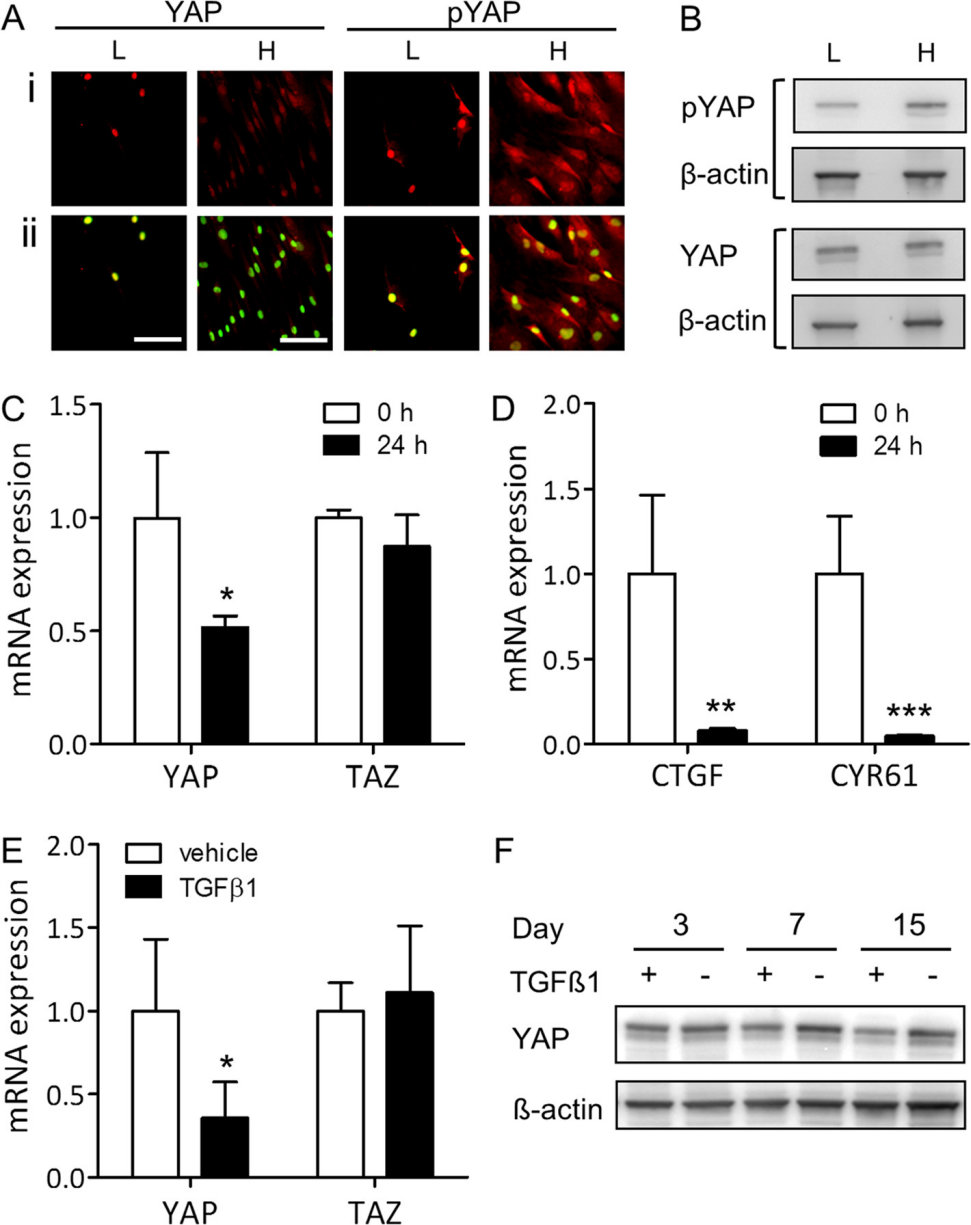
YAP and TAZ expression at high density and during chondrogenic differentiation of human synovial MSCs. **(A)** YAP and pYAP expression in human synovial membrane-derived (hSM-)MSCs in monolayer at low (L) and high (H) density detected by immunofluorescence staining, shown without (i) and with sytox green nuclear counterstain (ii). **(B)** YAP and pYAP expression in hSM-MSCs in monolayer at low (L) and high (H) density detected by western blotting with β-actin as loading control. **(C, D)** Expression of YAP and TAZ (C) and their target genes CTGF and CYR61 (D) in hSM-MSCs immediately prior to (0 h) or 24 h after plating in micromass culture, determined by quantitative RT-PCR. Data was normalised to GAPDH expression, and is shown as mean ± standard deviation (SD) (three donors) relative to pre-seeding (0 h) control. ^*^ *P* <0.05; ^**^ *P* <0.01; ^***^ *P* <0.001. **(E)** Expression of YAP and TAZ in hSM-MSCs after 6 days of treatment with 10 ng/ml TGF-β1 or vehicle only in micromass culture to induce chondrogenic differentiation, determined by quantitative RT-PCR. Data was normalised to GAPDH expression, and is shown as mean ± SD (five donors) relative to vehicle-treated control. ^*^ *P* <0.05. **(F)** Detection of YAP by western blotting during chondrogenic differentiation induced by TGF-β1 with detection of β-actin as loading control. MSC, mesenchymal stromal/stem cell; pYAP, phosphorylated YAP; RT-PCR, reverse transcription PCR; TAZ, transcriptional co-activator with PDZ-binding motif; TGF, transforming growth factor; YAP, Yes-associated protein.

Induction of chondrogenesis in MSCs requires high cell density mimicking embryonic mesenchymal condensation, for example by culturing cells in micromass. Co-transcriptional activity of YAP is typically downregulated at high cell density through phosphorylation at key amino acid residues, most notably Ser127, and sequestration in the cytosol and/or proteasomal degradation [10,17]. Indeed, YAP was predominantly nuclear in hSM MSCs cultured at low cell density but more phosphorylated and cytosolic at high cell density (Figure 1A and B). In addition to the post-translational regulation of YAP at high cell density, YAP mRNA levels were significantly decreased after 1 day of culture in micromass prior to initiation of TGF-β1 treatment (Figure 1C). Accordingly, the expression of the YAP downstream target genes CTGF and CYR61 was strongly decreased 1 day after plating in micromass (Figure 1D). YAP expression was further downregulated after 6 days of TGF-β1-induced chondrogenesis, as compared to vehicle-treated control cultures (Figure 1E). Similar results were obtained with hMSCs isolated from periosteum or bone marrow (Figure S1 in Additional file 1). Downregulation of YAP at the protein level in response to TGF-β1-induced chondrogenesis became apparent after 1 week (Figure 1F), and paralleled the induction of a chondrogenic phenotype as determined by alcian blue staining of glycosaminoglycans (Figure S2 in Additional file 2). In contrast to YAP, the expression of its paralogue TAZ in hSM-MSCs was not decreased either 1 day after plating in micromass (Figure 1C) or after 6 days of chondrogenic treatment (Figure 1E).

### Expression of human YAP transcript variants during chondrogenesis

**Figure 2:**
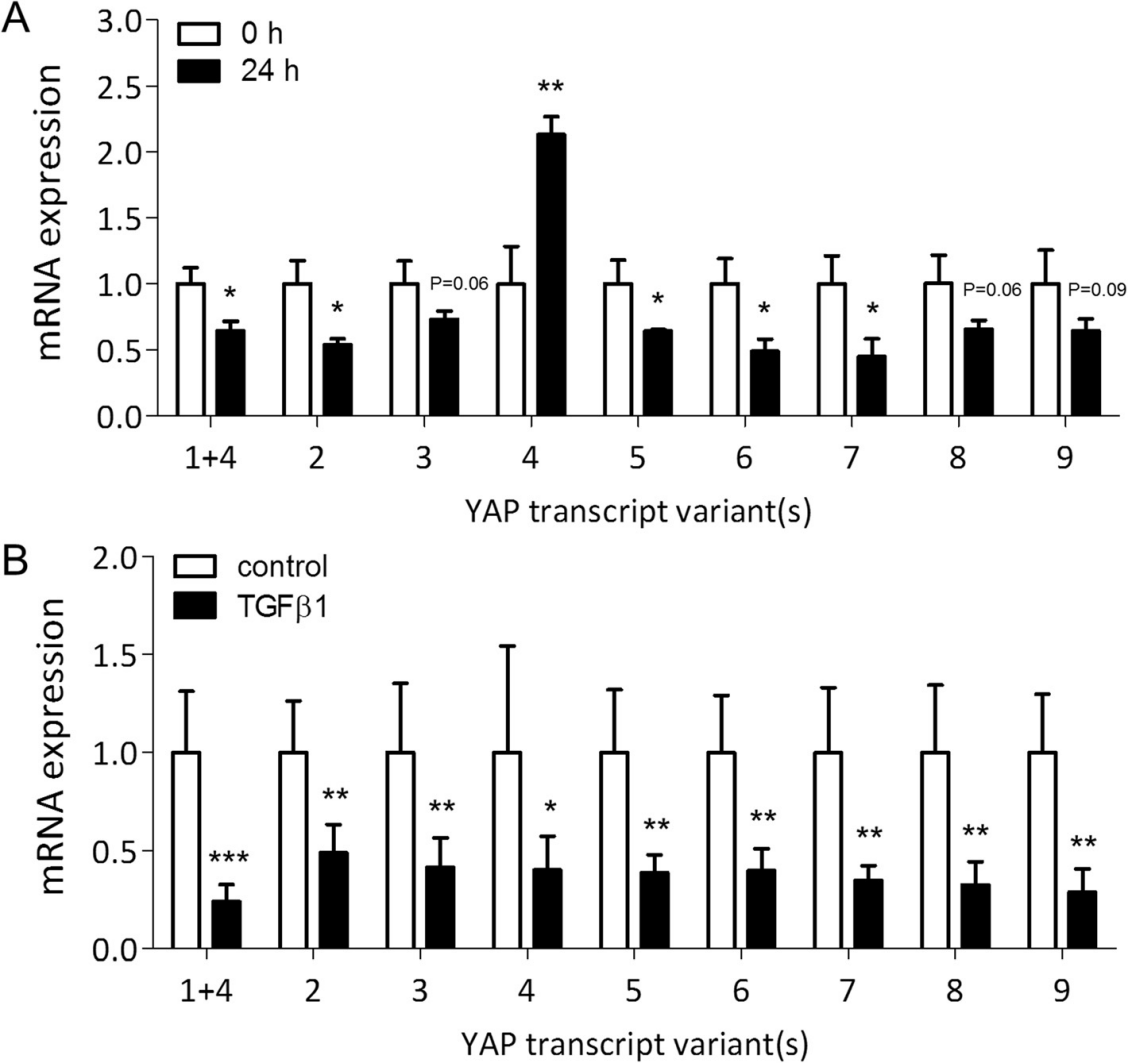
Expression of YAP transcript variants during micromass culture and chondrogenic differentiation of human synovial MSCs. **(A)** Expression of YAP transcript variants in human synovial membrane-derived (hSM)-MSCs immediately prior to (0 h) or 24 h after plating in micromass culture, shown as mean ± standard deviation (SD) (three donors) relative to pre-seeding (0 h) control. **(B)** Expression of YAP transcript variants in hSM-MSCs after 6 d of treatment with 10 ng/ml TGF-β1 or vehicle only in micromass culture, shown as mean ± SD (five donors) relative to vehicle-treated control. All data was normalised to GAPDH expression. ^*^ *P* <0.05; ^**^ *P* <0.01; ^***^ *P* <0.001. MSC, mesenchymal stromal/stem cell; TGF, transforming growth factor; YAP, Yes-associated protein.

Human YAP (hYAP) is subject to extensive alternative splicing, resulting in the existence of multiple transcript variants [30]. To determine whether the transcript variants of hYAP are subject to differential regulation of expression during chondrogenesis, we designed primers specific for the nine currently known hYAP transcripts (Table 1; primers for transcript variant 1 co-detect variant 4). Primer specificity was confirmed by gel electrophoresis of qPCR products, which revealed single bands of correct sizes (Figure S3 in Additional file 3). All transcript variants were found to be expressed by hSM-MSCs. After 1 day of micromass culture, transcripts 1 + 4, 2, 5, 6, and 7 were significantly decreased, while transcripts 3, 8, and 9 showed a trend towards decrease, compared to transcript levels immediately prior to plating in micromass (Figure 2A). In contrast, mRNA levels of transcript variant 4 were significantly higher (Figure 2A). After TGF-β1-induced chondrogenic differentiation for 6 days, all transcripts including variant 4 were downregulated compared to control cultures (Figure 2B).

### High YAP expression inhibits chondrogenic differentiation

**Figure 3:**
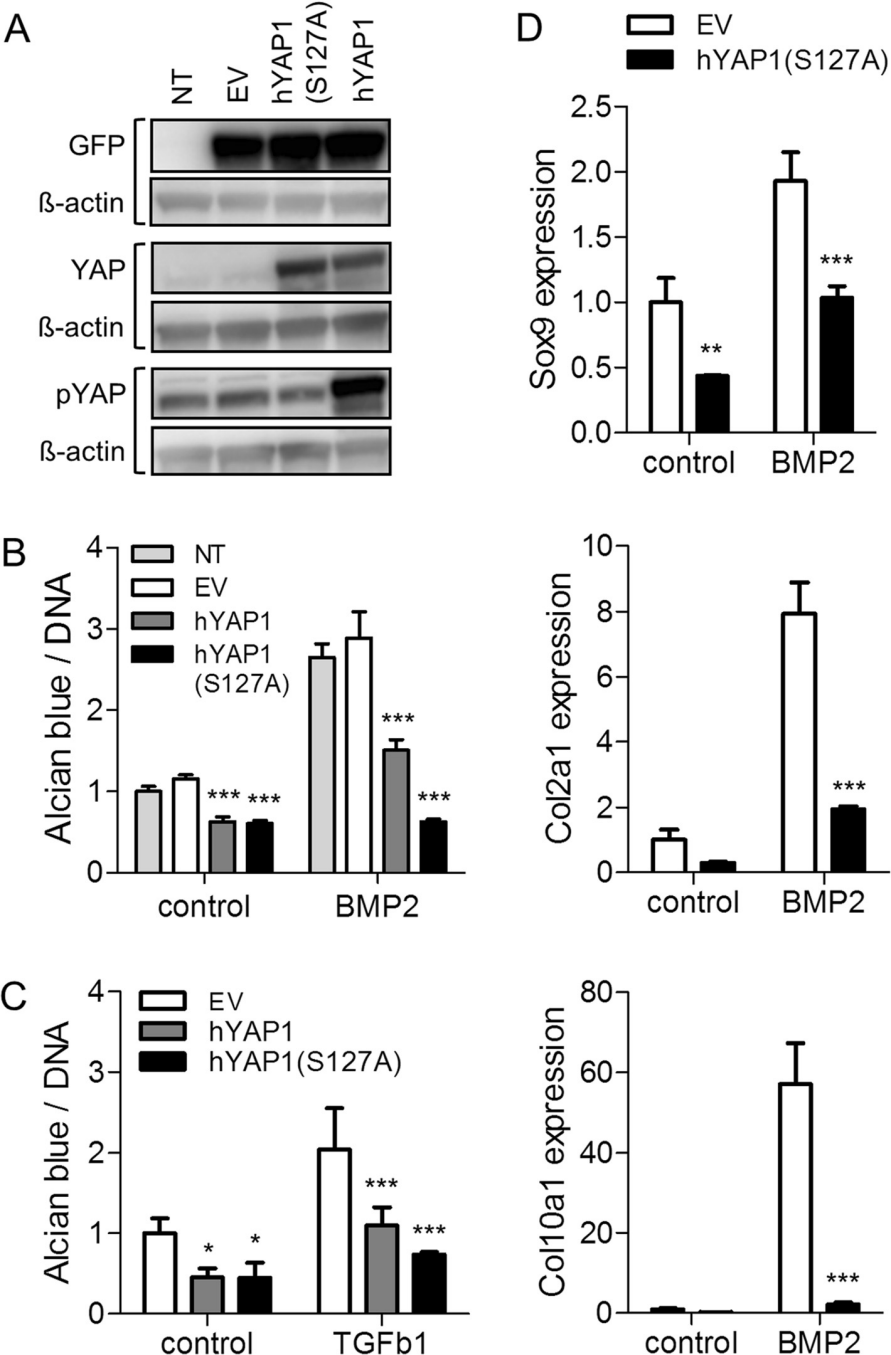
Effect of overexpression of YAP on chondrogenic differentiation of C3H10T1/2 MSCs. C3H10T1/2 cells were transduced with retrovirus encoding hYAP1 or hYAP1(S127A) and GFP separated from the hYAP1 gene by an IRES sequence. Cells transduced with empty vector (EV) retrovirus and/or cells left non-transduced (NT) served as controls. **(A)** Detection of YAP, pYAP, and GFP by western blotting. **(B-D)** Cells were treated with 300 ng/ml BMP-2 **(B, D)** or 10 ng/ml TGF-β1 **(C)** in micromass culture for 7 days to induce chondrogenic differentiation. **(B, C)** Whole-mount alcian blue staining followed by extraction and measurement of absorbance at 630 nm. Absorbance was normalised to DNA content determined from parallel micromass cultures. Data is shown as mean ± standard deviation (SD) (n = 4), and expressed relative to the NT **(B)** or EV controls **(C)**. **(D)** Chondrogenic marker expression by quantitative RT-PCR. Data is shown as mean ± SD (n = 3). ^**^ *P* <0.01; ^***^ *P* <0.001. BMP, bone morphogenetic protein; GFP, green fluorescent protein; MSC, mesenchymal stromal/stem cell; pYAP, phosphorylated YAP; RT-PCR, reverse transcription PCR; TGF, transforming growth factor; YAP, Yes-associated protein.

**Figure 4:**
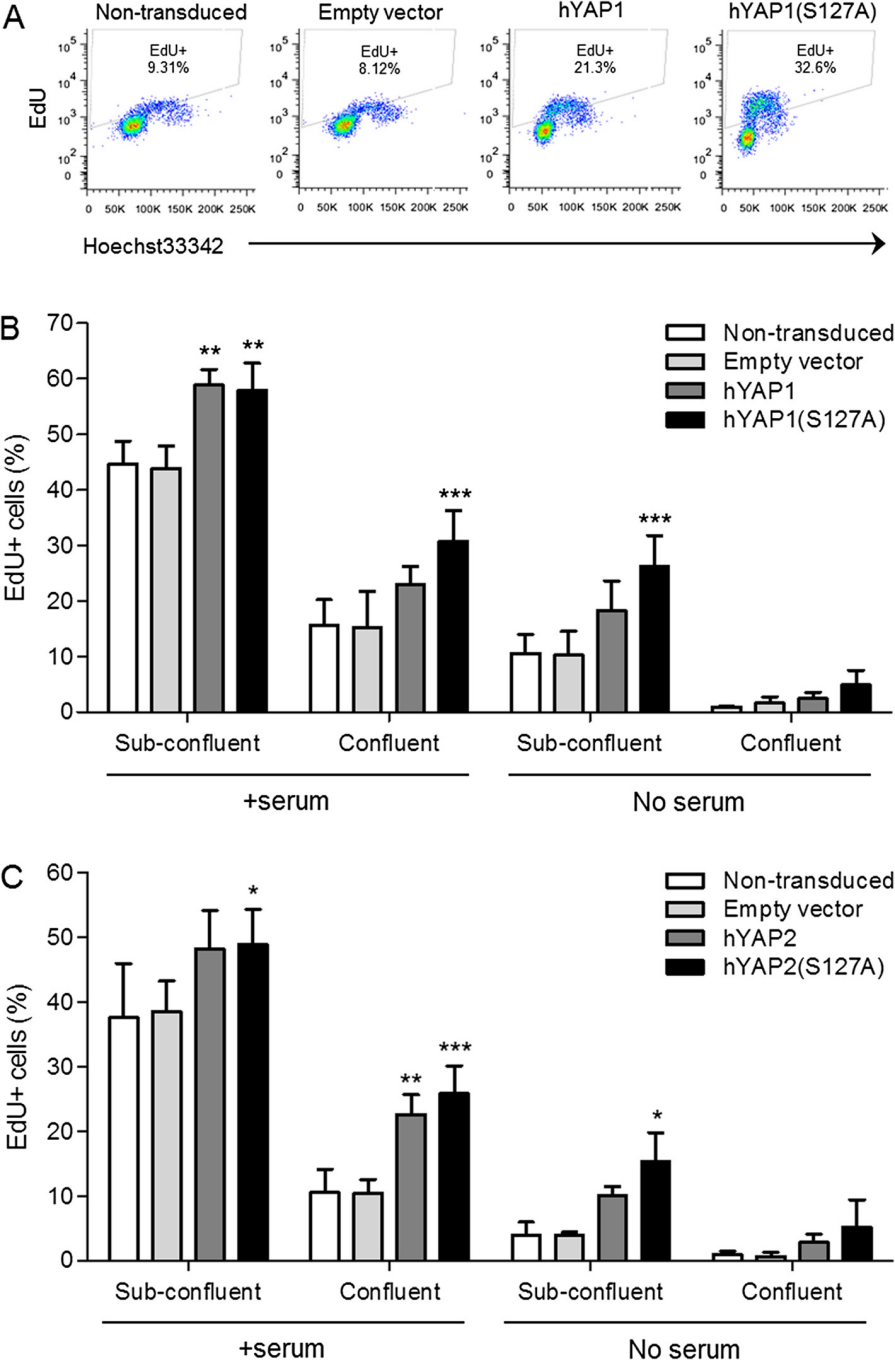
Effect of overexpression of YAP on cell proliferation. C3H10T1/2 cells were transduced with retrovirus encoding hYAP1 or hYAP1(S127A) **(A, B)** or hYAP2 or hYAP2(S127A) **(C)**. Cells transduced with empty vector retrovirus and cells left non-transduced served as controls. Cells cultured at sub-confluence or confluence were maintained for 24 h in the presence or absence of serum, then cell proliferation was determined by EdU nucleoside incorporation into replicating DNA, detected using the Click-iT system. **(A)** Representative flow cytometry plots of cells cultured at sub-confluence in the absence of serum. **(B, C)** Percentage of EdU+ cells quantified using FlowJo software. Data is shown as mean ± standard deviation (SD) (n = 3). ^*^ *P* <0.05; ^**^ *P* <0.01; ^***^ *P* <0.001. YAP, Yes-associated protein.

Downregulation of YAP during chondrogenesis suggested that YAP may be a negative regulator of chondrogenic differentiation in MSCs. To further investigate this, we elected to use the murine C3H10T1/2 MSCs were for their amenability to genetic manipulations. MSCs were retrovirally transduced with hYAP1 (transcript variant 5; [27]), constitutively active hYAP1(S127A), or empty vector. Expression of transgenes was confirmed by western blotting (Figure 3A). Alcian blue staining to detect glycosaminoglycan deposition revealed a partial inhibition of BMP-2-induced chondrogenesis in hYAP1-transduced cells and a complete inhibition in cells expressing the constitutively active hYAP1(S127A), as compared to EV-transduced cells (Figure 3B, Figure S4A in Additional file 4). Similar results were obtained when cells were transduced with the hYAP2 isoform (transcript variant 3; [27]) (Figure S4C and D in Additional file 4). Expression of the different transcript variants was confirmed by qRT-PCR using human-specific primers (Figure S5 in Additional file 5). Chondrogenesis induced by treatment with TGF-β1 was also impaired in hYAP1-transduced cells (Figure 3C, Figure S4B in Additional file 4). Inhibition of chondrogenesis was confirmed by significantly lower expression levels of the chondrocyte-lineage genes Sox9, Col2a1 and Col10a1 in hYAP1(S127A)-transduced cells compared to empty vector-transduced cells after **7** days of BMP-2 treatment (Figure 3D). By contrast, overexpression of hYAP1 or hYAP2 increased proliferation of C3H10T1/2 cells under all culture conditions tested (Figure 4). Constitutively active hYAP had a greater effect than wild-type hYAP under conditions of serum withdrawal or high confluence.

### High YAP expression impairs BMP signalling during chondrogenesis

**Figure 5:**
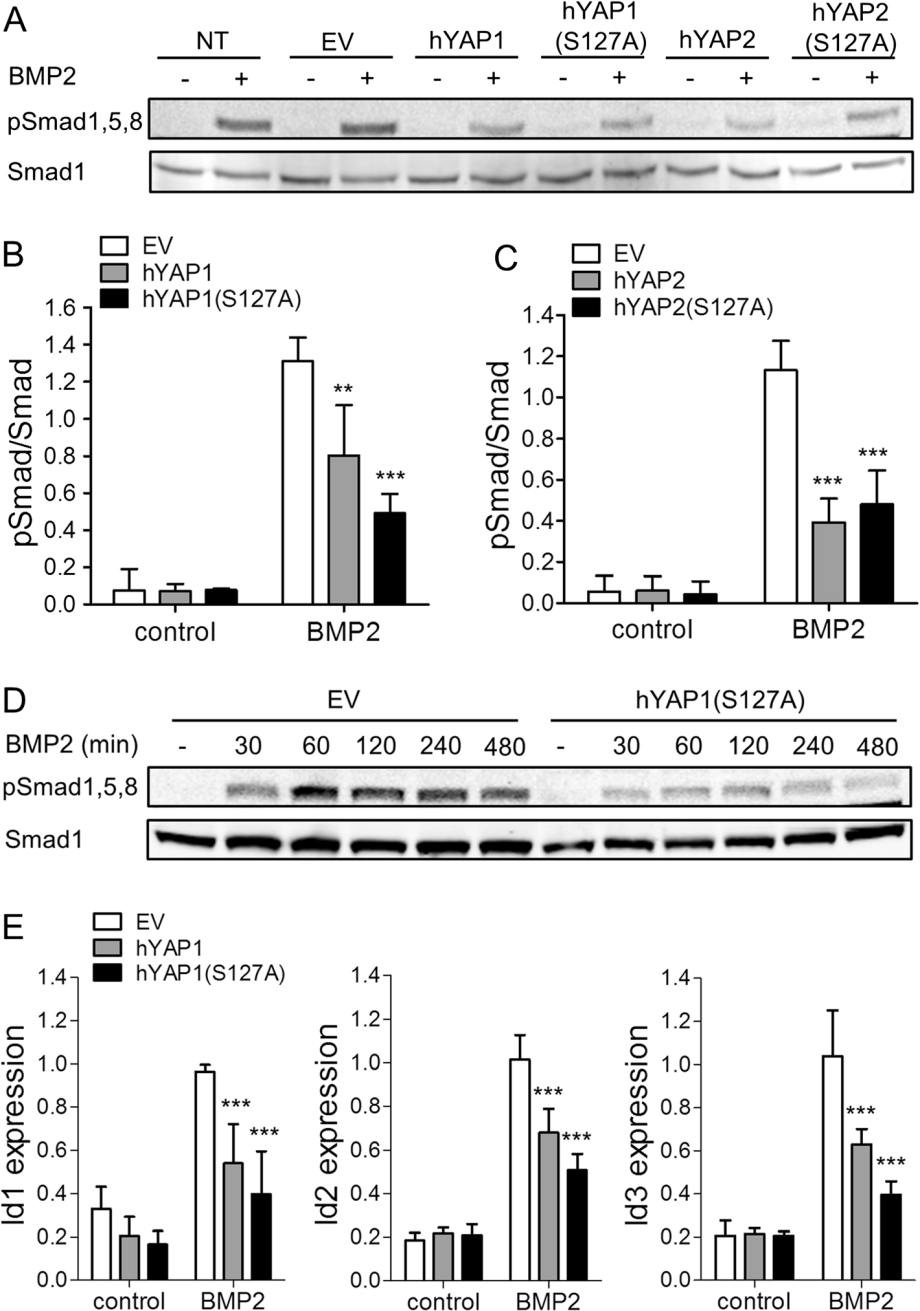
Effect of overexpression of YAP on BMP-2 signalling. C3H10T1/2 cells were transduced with retrovirus encoding hYAP1, hYAP1(S127A), hYAP2 or hYAP2(S127A), and GFP separated from the YAP gene by an IRES sequence. Cells transduced with empty vector (EV) retrovirus and/or cells left non-transduced (NT) served as controls. Cells were plated in micromass culture and the next day treated with 300 ng/ml BMP-2 or vehicle only. **(A)** pSmad1,5,8 and total Smad1 after 1 h of BMP2 treatment determined by western blotting. **(B, C)** Ratio of pSmad1,5,8/Smad1 after 1 h of BMP2 treatment shown as mean ± standard deviation (SD) (n = 3). ^**^ *P* <0.01; ^***^ *P* <0.001. **(D)** Time-course of BMP-2-induced pSmad1,5,8 and total Smad1. **(E)** Expression of the BMP target genes Inhibitor of differentiation (Id)1, Id2 and Id3 after 4 h of BMP-2 treatment determined by quantitative RT-PCR. Data was normalised to ACTB expression, and is shown as mean ± SD (n = 4). ^***^ *P* <0.001. BMP, bone morphogenetic protein; GFP, green fluorescent protein; pSmad, phosphorylated Smad; pYAP, phosphorylated YAP; RT-PCR, reverse transcription PCR; YAP, Yes-associated protein.

YAP has been reported to modulate BMP and TGF-β signalling through direct interactions with Smad proteins [31,32]. We therefore sought to determine whether the inhibition of BMP-2-induced chondrogenic differentiation of C3H10T1/2 MSCs transduced with hYAP was due to impaired BMP signalling responses, by detecting phosphorylation of Smad1,5,8 by western blotting. We analysed both hYAP1 (variant 5, containing 1 WW domain) and hYAP2 (variant 3, containing 2 WW domains) wild-type and S127A mutant forms. Both hYAP transcript variants decreased pSmad1,5,8 levels compared to the non-transduced and EV controls (Figure 5A-C). Time-course analysis indicated that pSmad1,5,8 levels were lower in hYAP1(S127A)-transduced cells compared to EV controls throughout the BMP-2 treatment period (Figure 5D). To determine whether the observed decrease in phosphorylation of Smad1,5,8 resulted in decreased Smad transcriptional activity, the expression of the BMP target genes Id1, Id2 and Id3 was determined by quantitative RT-PCR. While neither transcript variant of hYAP affected basal expression levels of Id genes, the upregulation of all three Id genes in response to BMP-2 treatment was significantly blunted in cells transduced with hYAP1 or hYAP1(S127A) (Figure 5E). Similar results were obtained with hYAP2 and hYAP2(S127A) (Figure S6 in Additional file 6).

### Yap expression during embryonic limb development

**Figure 6:**
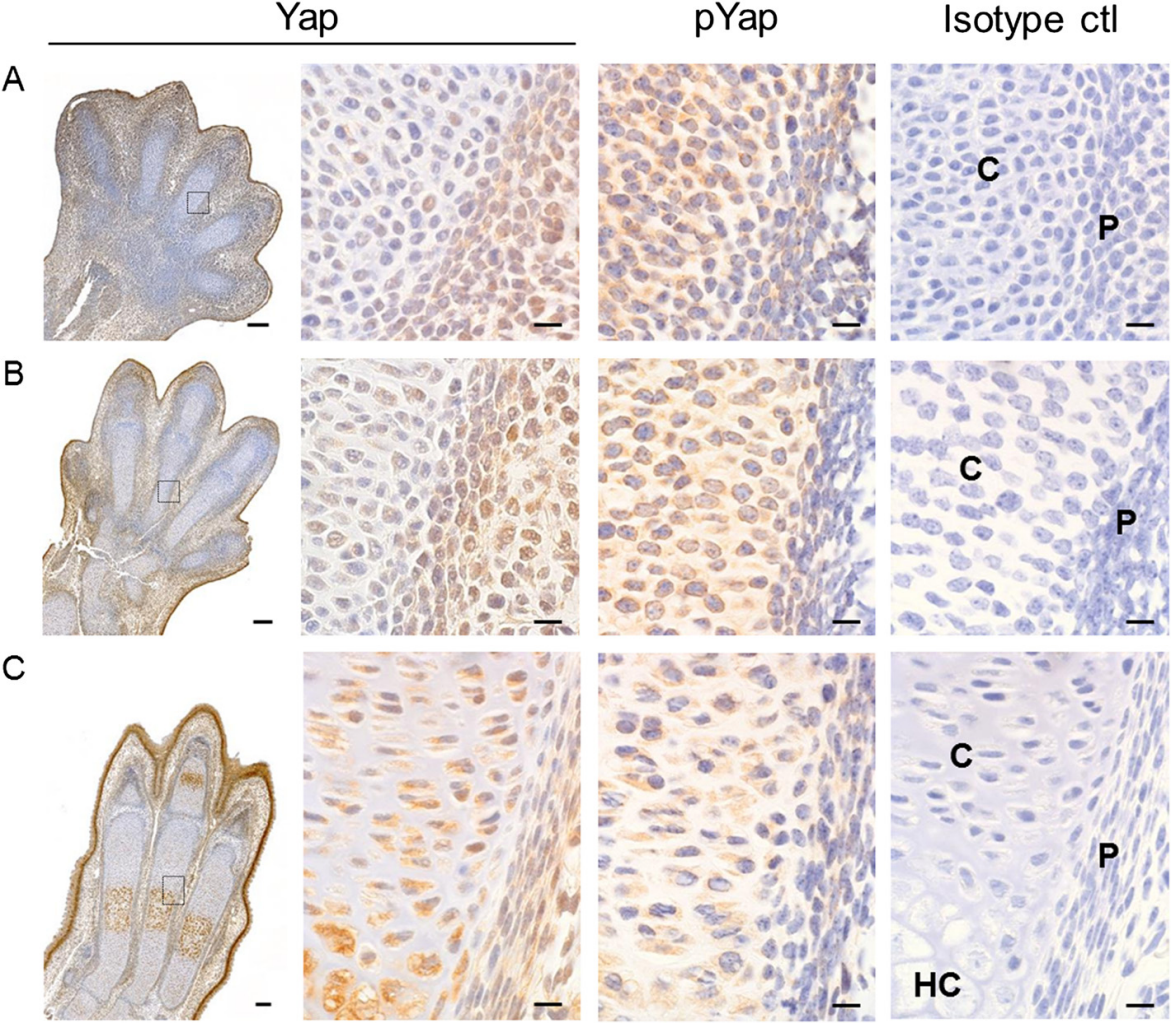
Yap and pYap expression during limb development. Consecutive histological sections from hindlimbs of mouse embryos at E13.5 **(A)**, E14.5 **(B)** and E16.0 **(C)** were immunohistochemically stained for Yap or pYap, or stained with an immunoglobulin G (IgG) isotype control antibody, and counterstained with haematoxylin. Results shown are representative of data from three embryos at each time point. Boxed area on left shows approximate location of higher magnification images on right. P: perichondrium, C: cartilage anlage. HC: hypertrophic cartilage. Bars indicate 100 μm (tile scan images on left; 20× objective) and 10 μm (higher magnification images on right; 100× oil objective). Images on far right are representative isotype controls for Yap **(A, C)** and pYap **(B)**. pYap, phosphorylated Yap; Yap, Yes-associated protein.

Finally, to gain insight in a possible role for YAP in physiological chondrogenesis *in vivo*, we investigated the expression of Yap and phosphorylated Yap during mouse limb development. Focusing on the phalanges of the hindlimbs, cells in the perichondrium showed a high degree of nuclear localisation of Yap at E13.5 (Figure 6A) when the phalanges are forming. In contrast, cells throughout the cartilage anlage showed low levels of nuclear Yap, while staining for cytosolic, phosphorylated Yap was more pronounced (Figure 6A). These differential expression patterns between the forming cartilage and perichondrium continued to be apparent at E14.5 (Figure 6B). These data show that Yap is present during embryonic cartilage formation with expression patterns that suggest high activity in mesenchymal cells and Yap phosphorylation (inactivation) in cells undergoing chondrogenic differentiation *in vivo*. At E16.0, when cartilage templates have fully formed and chondrocytes towards the centre of the phalanges are undergoing hypertrophy, Yap levels were increased in pre-hypertrophic chondrocytes and even higher in hypertrophic chondrocytes (Figure 6C), indicating upregulation of Yap during chondrocyte terminal differentiation.

## Discussion

The factors that determine whether a stem cell will proliferate or differentiate, and what differentiation lineage that cell will follow, are incompletely understood. The transcriptional co-factor YAP and its paralogue TAZ have recently emerged as key regulators of stem cell fate [33]. In bone marrow MSCs, expression of nuclear active YAP promotes osteogenic differentiation while adipogenesis requires low YAP activity [20]. Whether YAP plays a role in the regulation of chondrogenesis was not clear.

Chondrogenesis during embryonic development follows condensation of cells in the early limb bud to form a densely packed mesenchyme [34]. Analogous to this developmental process, chondrogenesis of MSCs *in vitro* is induced by high cell density culture (for example micromass culture, as originally developed by Ahrens *et al*. [35]). These are conditions under which YAP activity is typically low. Indeed, we found that YAP expression and activity was rapidly downregulated in MSCs at high cell density and in micromass culture, even before treatment with TGF-β to induce chondrogenesis was initiated, suggesting that downregulation of YAP at high cell density may prime cells for chondrogenesis. YAP was further downregulated during chondrogenic differentiation, as compared to non-chondrogenic control cultures.

The YAP gene in humans is subject to extensive alternative splicing, generating nine currently known transcript variants. Eight transcripts (termed variants 1, 2, 3, 5, 6, 7, 8, and 9) share a common start site and are generated by alternative splicing at exon 4 (which encodes the second WW domain), at the final 12 nucleotides of exon 5, and at exon 6 [30]. The ninth transcript (termed variant 4) has an alternative transcription start site located within exon 2 of the YAP gene (NCBI Ref Seq NM_001195045.1). To determine whether YAP transcript variants are subject to differential regulation in hMSCs during micromass culture and chondrogenesis, we designed transcript variant-specific primers. This revealed that in response to culture in high density micromass, the eight transcripts that share a common start site were all downregulated, while transcript variant 4 was upregulated. This is, to the best of our knowledge, the first demonstration of differential regulation of YAP transcript variants. Considering the different transcriptional start site, this suggests that variant 4 may be under the control of distinct promoter or enhancer elements. Future studies will be required to determine the transcriptional mechanisms underlying the differential regulation of variant 4 in response to cell density, and whether this isoform has distinct functions within the cell.

In contrast to YAP, expression of the YAP paralogue TAZ did not change following plating in micromass, or following subsequent chondrogenesis. We therefore focused our study on YAP. Of note, while YAP and TAZ frequently function redundantly and are often referred to together as YAP/TAZ [36], disparate regulation and function of YAP and TAZ have been reported previously, most notably in the modulation of myogenesis. While YAP is downregulated during myogenesis and high YAP activity inhibits myogenic differentiation of C2C12 myoblasts [37] and satellite cells [14], TAZ acts as a positive regulator of myogenesis *in vitro* and muscle regeneration after injury *in vivo* [38].

To determine whether low nuclear YAP is required for chondrogenic differentiation, we overexpressed either wild-type or a non-phosphorylatable mutant form of YAP in mouse C3H10T1/2 MSCs and subjected the cells to chondrogenic culture conditions. Forced YAP expression decreased chondrogenic differentiation, while mutant YAP almost completely abrogated chondrogenesis. Similar results were found whether we used BMP-2 or TGF-β1. By contrast, YAP overexpression increased proliferation, suggesting a role for YAP as a molecular switch between proliferation and chondrogenic differentiation in MSCs.

Findings from recent studies investigating a role for YAP and TAZ in modulating chondrogenesis in response to mechanical cues are consistent with our data. In cultured chondrocytes, high YAP activity under high fluid-flow shear stress or on stiff matrices correlated with loss of chondrocyte phenotype [21,39], with knockdown of YAP in cells grown on stiff matrices preserving chondrogenic marker expression [39]. Similarly, knockdown of YAP/TAZ in rat MSCs cultured for 4 weeks in chondrogenic medium in a microfluidic PLGA-nanofibre device resulted in a significant increase in the expression of chondrocyte-lineage markers [40]. In addition, knockout of membrane-type matrix metalloproteinase (MT-MMP) (MMP14) in mice, which leads to impaired pericellular matrix remodelling, resulted in decreased osteogenesis and increased adipogenesis and chondrogenesis of MSCs *in vitro* and *in vivo*, and these effects were reversed by forced expression of constitutively active YAP [41]. Thus, high YAP activity prevents chondrogenesis in MSCs under disparate experimental conditions but, prior to our study, the mechanism was not clear.

Here, we show that high YAP activity impairs chondrogenesis at least in part through suppressing Smad signalling, leading to decreased expression of the BMP target genes Id1, Id2 and Id3. Of interest, Id2 was shown to be required for BMP-induced chondrogenesis [42]. YAP was reported to modulate TGF-β/BMP signalling through direct interactions with R-Smads and inhibitory Smads. Upon BMP signalling in mouse embryonic stem cells (mESCs) YAP was recruited to the phosphorylated linker site of Smad1 and supported Smad1-dependent transcription of the target genes Id1 and Id2. Consequently, knockdown of YAP in mESCs suppressed Id gene expression. YAP can also interact with Smad7 and potentiate its inhibitory action on Smad signalling. Of note, the interaction of YAP with inhibitory Smads only requires the first WW domain (present in both YAP1 and YAP2), while interaction of YAP with R-Smads requires both WW domains [32]. The observation that, in our experiments, both hYAP1 (containing 1 WW domain) and hYAP2 (containing 2 WW domains) inhibited BMP-2 signalling is thus supportive of a dominant role for inhibitory Smad interactions. The exact mechanism, however, remains to be clarified.

We focused on BMP-2 signalling as we observed a more reliable and robust chondrogenic response of C3H10T1/2 MSCs with BMP-2 compared to TGF-β1, but it is likely that YAP functions in a similar way to dampen TGF-β1-induced chondrogenesis since YAP has been reported also to interact with and modulate Smad2,3 signalling [43,44].

Finally, to determine whether YAP may be associated with the regulation of chondrogenesis *in vivo*, we analysed Yap and pYap abundance during mouse embryonic limb development. While Yap was mostly nuclear in cells in the perichondrium, cells of the cartilage anlage showed high levels of phosphorylated, cytosolic Yap. These findings are consistent with our *in vitro* observations that YAP inhibits chondrogenesis. Of note, Yap expression was increased again during chondrocyte terminal differentiation, suggesting that Yap may play a role in chondrocyte hypertrophy.

## Conclusions

Taken together, our findings show that YAP is a negative regulator of chondrogenesis, and that YAP functions at least in part through repressing chondrogenic signalling while activating proliferative activity. Targeted modulation of YAP function in MSCs, for example through appropriate cues induced by a biomaterial and/or pharmacological inhibitors, may be a novel strategy to control MSC proliferation versus lineage specification for the effective repair of articular cartilage following injury or in OA.

## Additional files

Additional file 1: Figure S1. YAP expression during chondrogenic differentiation of human periosteal and bone marrow MSCs. YAP expression was determined by quantitative RT-PCR in human periosteal (A) and bone marrow MSCs (B) after 6 d of treatment with 10 ng/ml TGF-ß1 in micromass culture to induce chondrogenic differentiation. Data was normalised to GAPDH expression, and is shown as mean ± SD of five donors (A) or two donors (B) relative to control. ^*^ *P* <0.05 (no statistical analysis was performed on data in (B) due to low number of donors). Additional file 2: Figure S2. Time-course of chondrogenic differentiation of human synovial MSCs in micromass culture. Cells were treated with 10 ng/ml TGF-β1, or left untreated (control), in micromass culture for up to 21 days. Chondrogenesis was detected by whole-mount alcian blue staining followed by extraction and measurement of absorbance at 630 nm. Data are shown as mean ± SD (n = 3). Additional file 3: Figure S3. Detection of YAP transcript variants in human synovial MSCs. YAP transcript variants were detected by qRT-PCR followed by agarose gel electrophoresis using primers specific for individual YAP transcript variants. Band sizes correspond to expected amplicon sizes (see Table 1). Additional file 4: Figure S4. Effect of overexpression of hYAP variants on chondrogenic differentiation of C3H/10T1/2 cells. C3H10T1/2 cells were transduced with hYAP1 (A, B) or hYAP2 variants (C, D) and treated with 300 ng/ml BMP-2 (A, C, D) or 10 ng/ml TGF-β1 (B) in micromass culture for 7 days to induce chondrogenic differentiation. (A, B, C) Representative images of whole-mount alcian blue-stained micromasses. (D) Absorbance at 630 nm of extracted alcian blue dye. Data is shown as mean ± SD (n = 2). Additional file 5: Figure S5. Detection of hYAP in C3H10T1/2 cells transduced with retrovirus encoding different hYAP variants, or empty vector control. hYAP was detected by qRT-PCR followed by agarose gel electrophoresis using primers specific for human YAP that detect all transcript variants (hYAP-Fw4 and hYAP-Rev6; see Methods). Band sizes correspond to expected amplicon sizes for YAP1 (variant 5; 504 bp) and YAP2 (variant 3; 618 bp). Additional file 6: Figure S6. Effect of overexpression of hYAP2 on BMP target gene expression. C3H10T1/2 cells were transduced with retrovirus encoding hYAP2 or hYAP2(S127A). Cells transduced with empty vector (EV) retrovirus and/or cells left non-transduced (NT) served as controls. Cells were plated in micromass culture and the next day treated with 300 ng/ml BMP-2 or vehicle only. Expression of the BMP target genes Inhibitor of differentiation (Id)1, Id2 and Id3 was determined after 4 h of BMP-2 treatment by quantitative RT-PCR. Data was normalised to ACTB expression, and is shown as mean ± SD (n = 4) relative to BMP-2-treated NT controls. ^***^ *P* <0.001.

## Abbreviations

ANOVA: analysis of variance
BCA: bicinchoninic acid
BMP: bone morphogenetic protein
BSA: bovine serum albumin
ECM: extracellular matrix
EDTA: ethylenediaminetetraacetic acid
EGTA: ethylene glycol tetraacetic acid
DMEM: Dulbecco’s modified Eagle’s medium
FBS: foetal bovine serum
GFP: green fluorescent protein
HRP: horseradish peroxidase
hSM: human synovial membrane
IHC: immunohistochemistry
ITS: insulin, transferrin, sodium selenite
mESCs: mouse embryonic stem cells
MSC: mesenchymal stromal/stem cell
MT-MMP: membrane-type matrix metalloproteinase
Na_3_Vo: sodium orthovanadate
NaF: sodium fluoride
OA: osteoarthritis
PBS: phosphate-buffered saline
PCR: polymerase chain reaction
pSmad: phosphorylated Smad
pYAP: phosphorylated YAP
qPCR: quantitative PCR
R-Smad: receptor-activated Smad
RT-PCR: reverse transcription PCR
SDS: sodium dodecyl sulphate
TAZ: transcriptional co-activator with PDZ-binding motif
TGF: transforming growth factor
YAP: Yes-associated protein
αMEM: alpha minimum essential medium
Id: Inhibitor of DNA binding/differentiation
