# Supplementary figures and images for: Yes-associated protein (YAP) is a negative regulator of chondrogenesis in mesenchymal stem cells
Source: Arthritis Res Ther. 2015 May 30;17(1):147. doi: 10.1186/s13075-015-0639-9 (PMC4449558; doi:10.1186/s13075-015-0639-9)

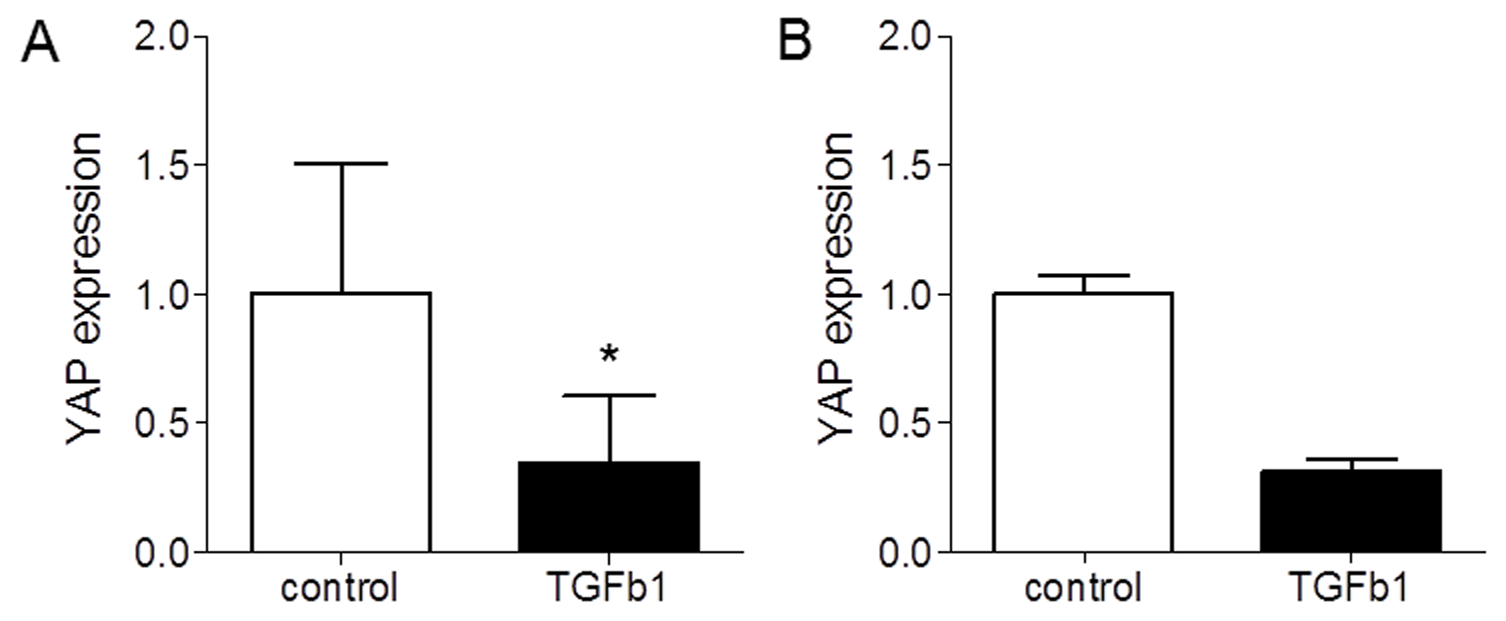

Supplement: Additional file 1: Figure S1. — YAP expression during chondrogenic differentiation of human periosteal and bone marrow MSCs. YAP expression was determined by quantitative RT-PCR in human periosteal (A) and bone marrow MSCs (B) after 6 d of treatment with 10 ng/ml TGF-ß1 in micromass culture to induce chondrogenic differentiation. Data was normalised to GAPDH expression, and is shown as mean ± SD of five donors (A) or two donors (B) relative to control. * P <0.05 (no statistical analysis was performed on data in (B) due to low number of donors). [file 13075_2015_639_MOESM1_ESM.tiff]

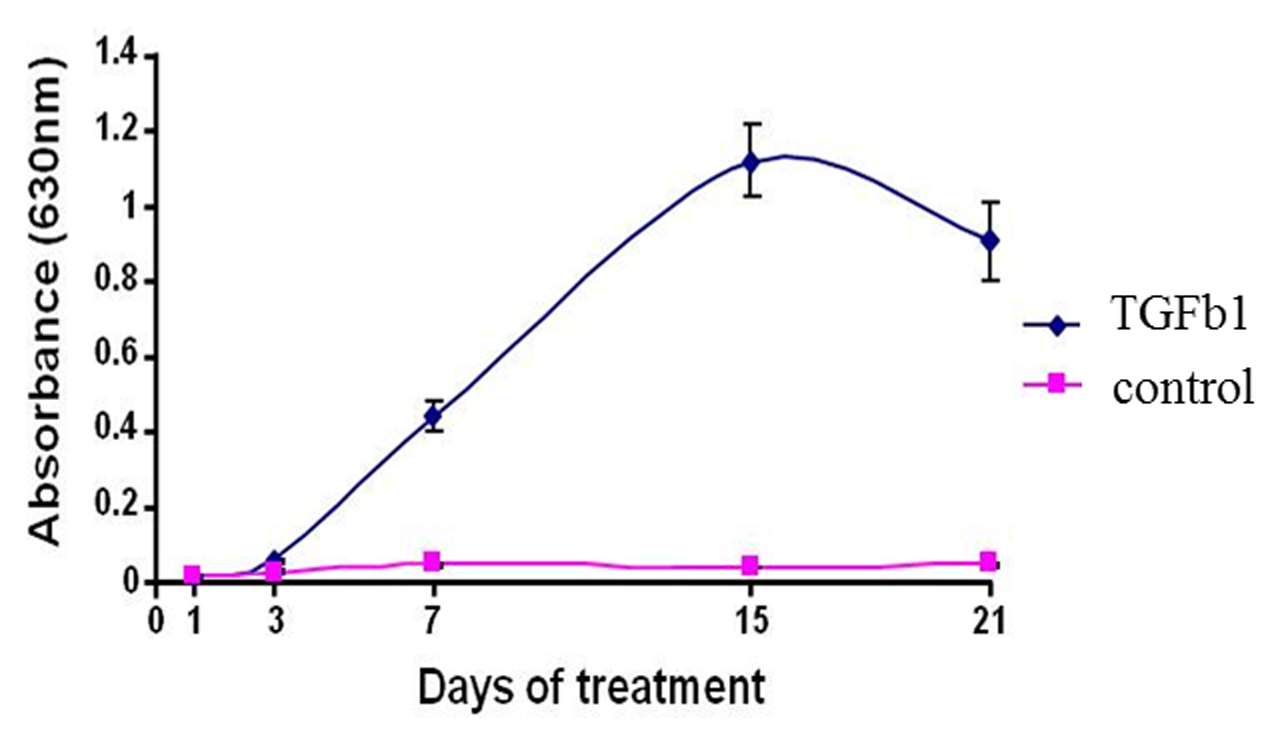

Supplement: Additional file 2: Figure S2. — Time-course of chondrogenic differentiation of human synovial MSCs in micromass culture. Cells were treated with 10 ng/ml TGF-β1, or left untreated (control), in micromass culture for up to 21 days. Chondrogenesis was detected by whole-mount alcian blue staining followed by extraction and measurement of absorbance at 630 nm. Data are shown as mean ± SD (n = 3). [file 13075_2015_639_MOESM2_ESM.tiff]

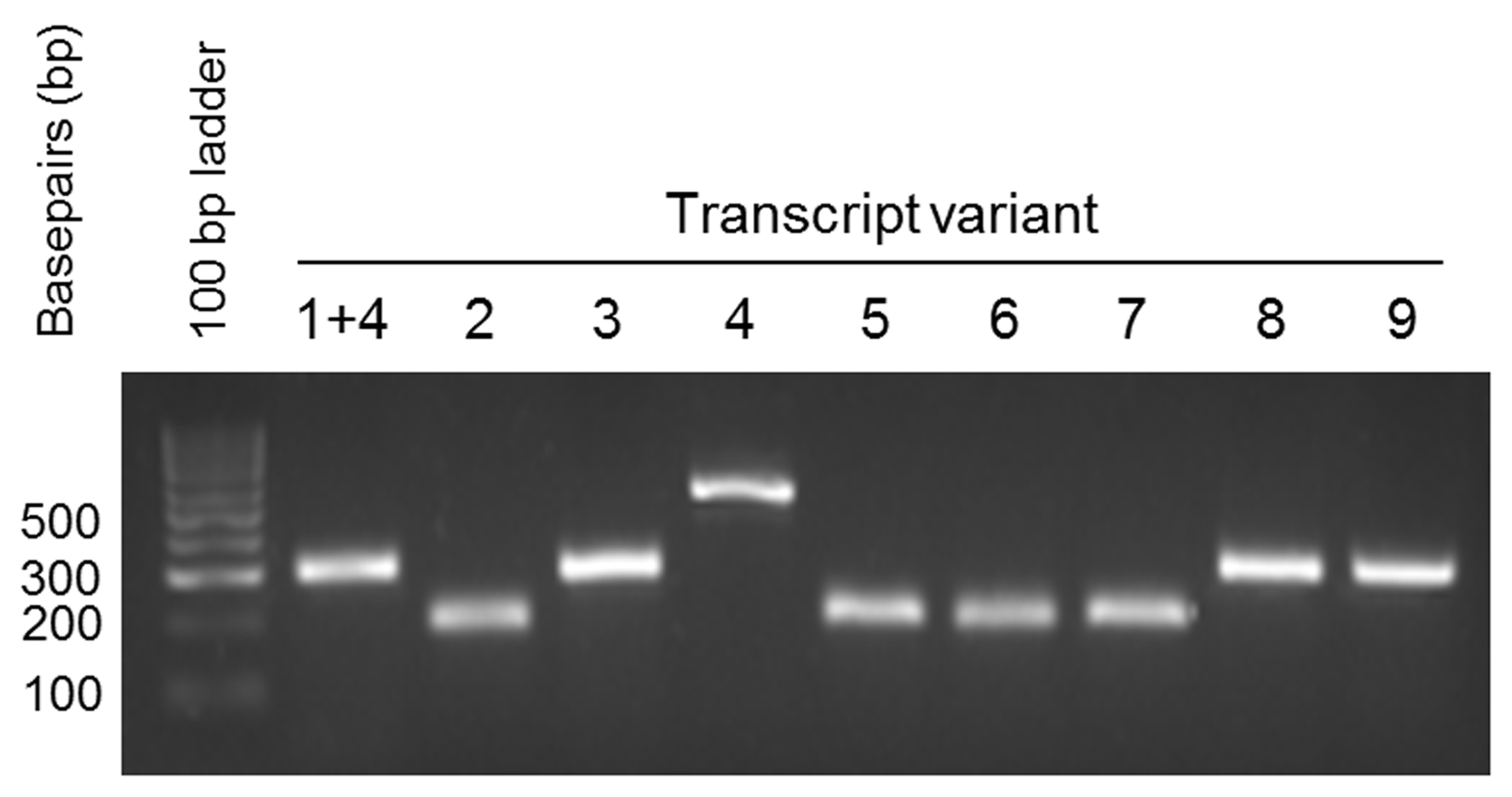

Supplement: Additional file 3: Figure S3. — Detection of YAP transcript variants in human synovial MSCs. YAP transcript variants were detected by qRT-PCR followed by agarose gel electrophoresis using primers specific for individual YAP transcript variants. Band sizes correspond to expected amplicon sizes (see Table 1). [file 13075_2015_639_MOESM3_ESM.tiff]

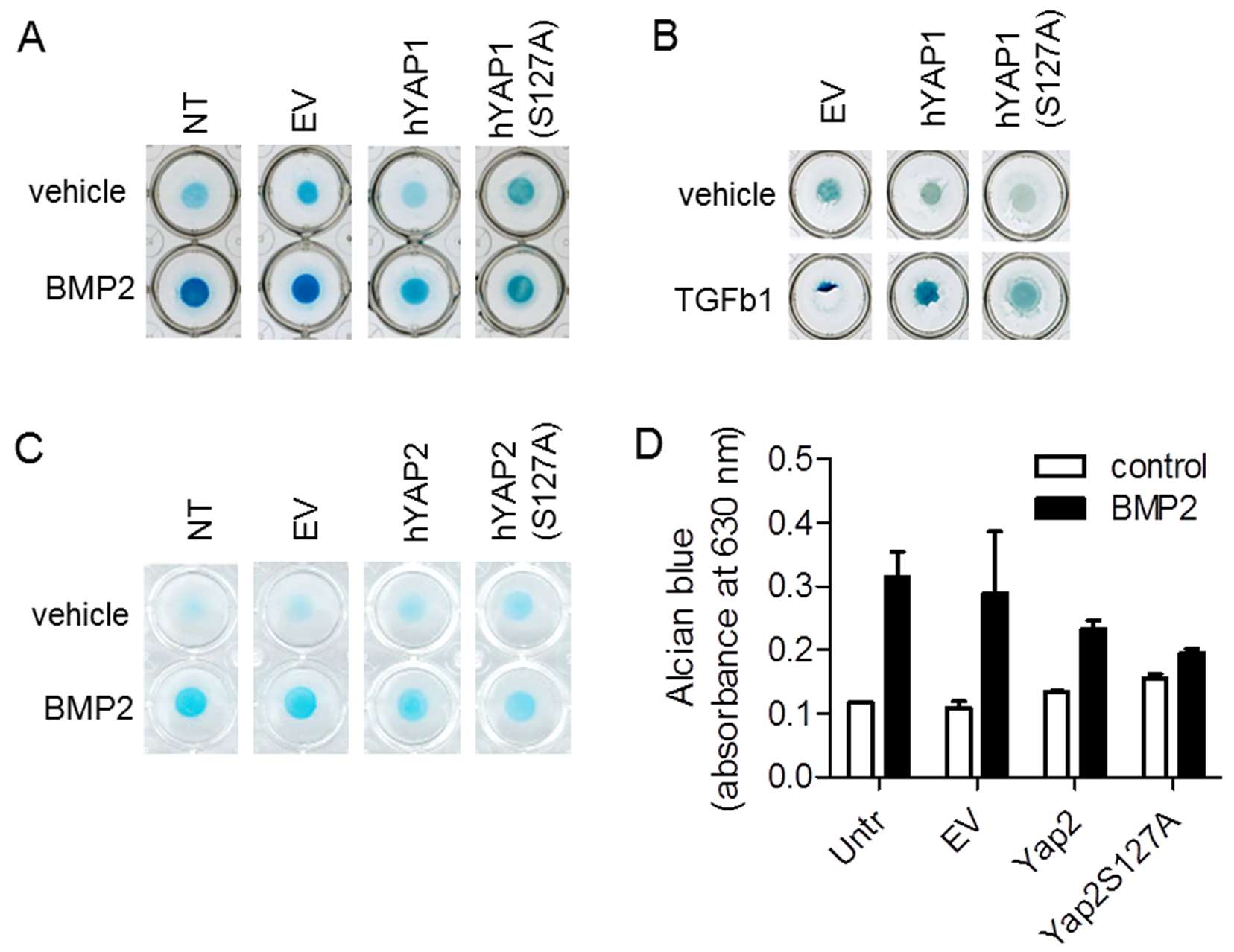

Supplement: Additional file 4: Figure S4. — Effect of overexpression of hYAP variants on chondrogenic differentiation of C3H/10T1/2 cells. C3H10T1/2 cells were transduced with hYAP1 (A, B) or hYAP2 variants (C, D) and treated with 300 ng/ml BMP-2 (A, C, D) or 10 ng/ml TGF-β1 (B) in micromass culture for 7 days to induce chondrogenic differentiation. (A, B, C) Representative images of whole-mount alcian blue-stained micromasses. (D) Absorbance at 630 nm of extracted alcian blue dye. Data is shown as mean ± SD (n = 2). [file 13075_2015_639_MOESM4_ESM.tiff]

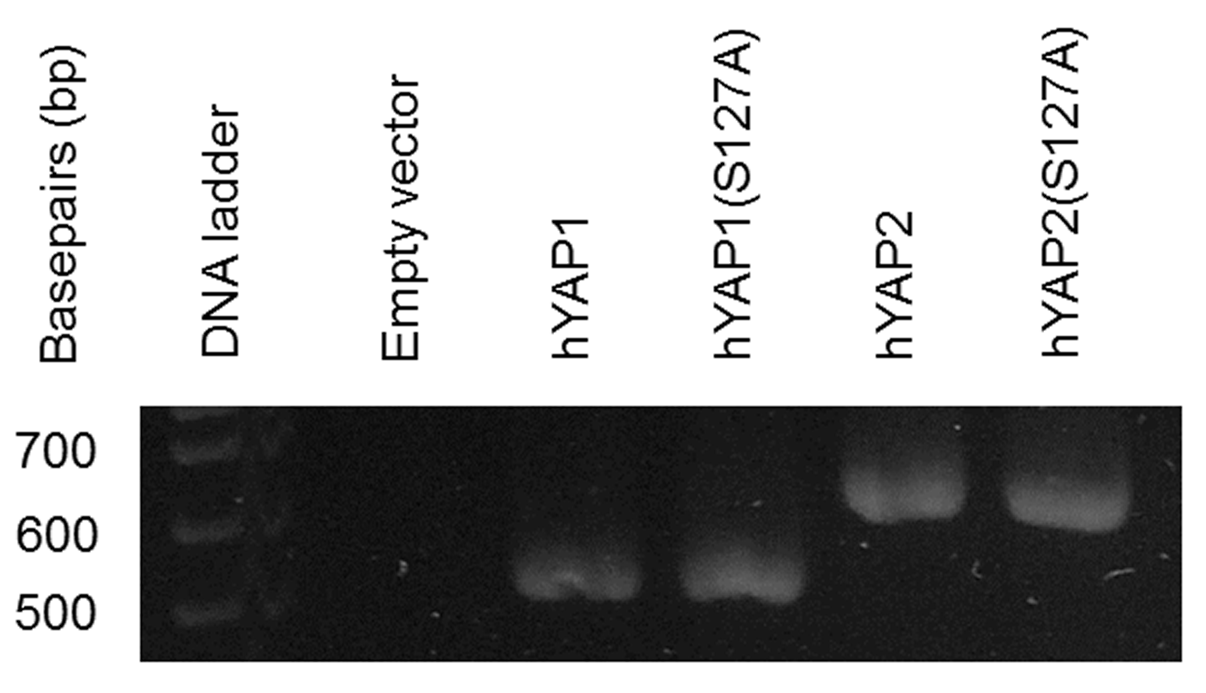

Supplement: Additional file 5: Figure S5. — Detection of hYAP in C3H10T1/2 cells transduced with retrovirus encoding different hYAP variants, or empty vector control. hYAP was detected by qRT-PCR followed by agarose gel electrophoresis using primers specific for human YAP that detect all transcript variants (hYAP-Fw4 and hYAP-Rev6; see Methods). Band sizes correspond to expected amplicon sizes for YAP1 (variant 5; 504 bp) and YAP2 (variant 3; 618 bp). [file 13075_2015_639_MOESM5_ESM.tiff]

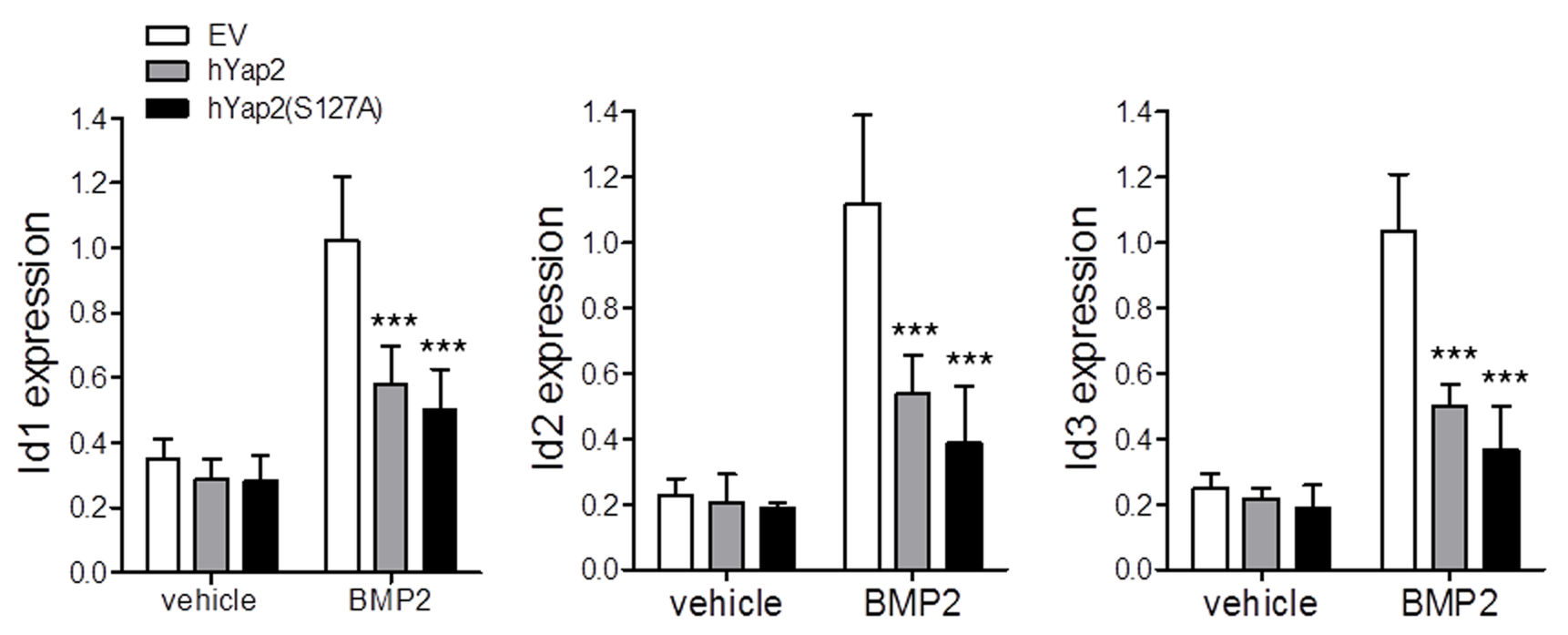

Supplement: Additional file 6: Figure S6. — Effect of overexpression of hYAP2 on BMP target gene expression. C3H10T1/2 cells were transduced with retrovirus encoding hYAP2 or hYAP2(S127A). Cells transduced with empty vector (EV) retrovirus and/or cells left non-transduced (NT) served as controls. Cells were plated in micromass culture and the next day treated with 300 ng/ml BMP-2 or vehicle only. Expression of the BMP target genes Inhibitor of differentiation (Id)1, Id2 and Id3 was determined after 4 h of BMP-2 treatment by quantitative RT-PCR. Data was normalised to ACTB expression, and is shown as mean ± SD (n = 4) relative to BMP-2-treated NT controls. *** P <0.001. [file 13075_2015_639_MOESM6_ESM.tiff]
